# A risk score model of 30-day readmission in ulcerative colitis after colectomy or proctectomy

**DOI:** 10.1038/s41424-018-0039-y

**Published:** 2018-08-15

**Authors:** Lindsay A. Sobotka, Syed G. Husain, Somashekar G. Krishna, Alice Hinton, Ravi Pavurula, Darwin L. Conwell, Cheng Zhang

**Affiliations:** 1 Department of Internal Medicine, The Ohio State University Wexner Medical Center, Columbus, OH USA; 2 Department of Surgery, Division of Colon and Rectal Surgery, The Ohio State University Wexner Medical Center, Columbus, OH USA; 3 Division of Gastroenterology, Hepatology and Nutrition, The Ohio State University Wexner Medical Center, Columbus, OH USA; 4 Division of Biostatistics, College of Public Health, The Ohio State University, Columbus, OH USA; 5 Department of Gastroenterology, Springfield Regional Medical Center, Mercy Health, Springfield, OH USA

## Abstract

**Introduction:**

The Center for Medicare and Medicaid Services established 30-day readmission rate as a key metric in measuring high-value, cost-conscious care; therefore, our aim is to develop a risk score for 30-day readmission in ulcerative colitis (UC) patients undergoing colectomy or proctectomy.

**Methods:**

This study used data from the American College of Surgeons National Surgical Quality Improvement Program (ACS NSQIP) participant user file (2011–2015). Patients with UC undergoing colectomy or proctectomy were identified using ICD-9, 10, and CPT codes. Stepwise multivariate analyses were used to determine risk factors associated with readmission including pre-operative conditions, laboratory results, operative variables, and post-operative complications. For readmission risk score assessment, a weighted logistic regression model was built and validated using ACS NSQIP 2011–2014 and 2015 data, respectively.

**Results:**

A total of 4797 patients were included with 963 (20%) patients readmitted within 30 days. Potentially modifiable risk factors included deep vein thrombosis, pulmonary embolism, renal insufficiency, wound infection, urinary tract infection, sepsis/septic shock, and pre-existing congestive heart failure. Ten percent of patients with a risk score between 0 and 9 were readmitted, 18.5% with a score between 10 and 19, 52.2% with a score between 20 and 29, and 59.6% in patients with a risk score >29.

**Conclusions:**

Multiple potentially preventable risk factors are associated with 30-day readmission following colectomy or proctectomy in UC patients. Higher risk scores are associated with increased risk of unplanned readmission.

## Introduction

Unplanned 30-day hospital readmission is an economic burden on the healthcare system, with an estimate cost to Medicare around $17.4 billion a year. Patients readmitted after colectomy or proctectomy are subject to a high mortality rate and increased complications^1,2^. In an effort to reduce readmission rates, the Center for Medicare and Medicaid Services (CMS) instituted a new policy in 2012, which established the Hospital Readmission Reduction Program. This program required CMS to reduce payment to hospitals that have a higher readmission rate than the national average, thus establishing readmission rate as a key factor in performing high-value, cost-conscious care^3^. Thirty-day readmission and mortality rates for specific diseases are now considered public knowledge in order to increase quality of care performed during inpatient admissions and to assist patients in selecting a hospital in which they would prefer to receive care^4^.

Ulcerative colitis (UC) is a chronic, relapsing disease with a prevalence of 238 per 100,000 patients per year in the United States^5^. Due to medically refractory disease, chronic corticosteroid use, untoward side effects of medication, dysplasia, or colorectal cancer, ~5–20% of UC patients undergo colectomy or proctectomy within 10 years of being diagnosed^5–7^. After undergoing colectomy or proctectomy, patients are at an increased risk for post-operative complications with 17–32% of patients having an unplanned readmission within 30 days^8–10^. Previous studies have evaluated factors associated with readmission, and while some have not determined any significant predictors, other studies have found proctocolectomy with ileal pouch-anal anastomosis (IPAA) and diverting ileostomy, age, and post-operative complications to be associated with readmission. However, these studies were all conducted on small patient populations at single institutions^8,10,11^.

Thus, we sought to determine the 30-day readmission rate and associated predictors of readmission in UC patients undergoing colectomy or proctectomy utilizing a large national clinical database. We hypothesized that many risk factors including pre-operative conditions, type of surgery, and post-operative complications would impact 30-day readmission rates.

## Materials and methods

### Data source

Data were obtained from the American College of Surgeons National Surgical Quality Improvement Program (ACS NSQIP) patient user file between 2011 and 2015. The ACS NSQIP collects reliable and validated information by trained and audited surgical reviewers at ~700 hospitals across the country. Data from the pre-operative period through 30-day post operation is collected using an 8th-day sample methodology in order to ensure that cases have an equal chance of being selected. Information obtained for each patient included patient demographics, laboratory values, medications, comorbidities, and 30-day post-operative outcomes and complications for patients undergoing a variety of surgeries^12,13^. Missing information from the user file during the study period is outlined in Supplementary Table 1.

### Study population and definition of variables

The study population included adult patients with a diagnosis of UC that underwent colectomy or proctectomy between 2011 and 2015. These patients were selected with the use of International Classification of Disease (ICD-9 and 10) codes for UC (556.xx and K51.xx, respectively) and Current Procedural Terminology (CPT) codes for colectomy or proctectomy. We excluded patients who died during the hospitalization or were hospitalized for >30 days.

#### Definition of pre-operative comorbidities, medications, and laboratory values

Pre-operative comorbidities included obesity, diabetes mellitus, severe chronic obstructive pulmonary disease (COPD), ascites, congestive heart failure within 30 days before surgery, hypertension requiring medication, acute renal failure, corticosteroid use for chronic conditions, >10% loss of body weight in the 6 months before surgery, bleeding disorders, anemia requiring transfusion prior to surgery, tobacco abuse within the year before surgery, and American Society of Anesthesiology (ASA) classification.

Pre-operative laboratory values included aspartate transaminase, hematocrit, albumin, and alkaline phosphatase.

#### Definition of colectomy or proctectomy and other surgical characteristics

Colectomy or proctectomy was defined as one of the following procedures: abdominal colectomy with end ileostomy (CPT codes: 44150 and 44210), proctocolectomy and end ileostomy (CPT codes: 44155 and 44212), proctocolectomy with IPAA creation and diverting ileostomy (CPT codes: 44157, 44158, and 44211), and proctectomy, which included proctectomy with combined abdominal perineal pull-through with creation of colonic reservoir and diverting enterostomy (CPT codes: 45119 and 45397), and proctectomy, partial with rectal mucosectomy, ileoanal anastomosis, and creation of ileal reservoir, with or without loop ileostomy (CPT code: 45113). Although proctectomy with combined abdominal perineal pull-through with creation of colonic reservoir and diverting enterostomy (CPT codes: 45119 and 45397) is not a common surgery for UC, many hospital coders including our institution coders often used it to code proctectomy with IPAA creation. In order to maximally capture patients, we included CPT codes, 45119 and 45397. Other surgical characteristics included laparoscopic versus open surgery, operation time, emergent versus non-emergent surgery, length of stay (LOS) associated with index surgery, and return to the operating room during the same admission.

#### Definition of post-surgical complications

Post-operative complications included surgical site infection (defined as superficial surgical site infection, deep incisional surgical site infection, and organ space surgical site infection), wound disruption, pneumonia, pulmonary embolism, renal insufficiency, urinary tract infection (UTI), cerebral vascular accident with neurologic deficits, cardiac arrest requiring cardiopulmonary resuscitation or myocardial infarction, bleeding transfusions, deep vein thrombosis (DVT) or thrombophlebitis, and sepsis or septic shock.

### Statistical analysis

SAS 9.4 (SAS Institute, Cary, NC) was used to perform all analyses. Continuous data were summarized with means and standard deviations and differences between groups were evaluated with *t* tests; log-transformations were employed where necessary prior to performing the *t* test. *χ*^2^-tests and Fisher exact tests were used to determine differences in categorical, as appropriate. Using NSQIP data from 2011 through 2014 as a developmental dataset logistic regression model was fit for 30-day readmission to evaluate factors association with readmission. Terms included in the model were determined through stepwise selection. This logistic regression model, with all continuous variables dichotomized, was used to construct the risk score for readmission. The coefficients, multiplied by 10 and rounded to the nearest integer, provided the weights used in the score for each of the risk factors. The fit of the model developed with NSQIP data from 2011 to 2014 was externally validated with NSQIP 2015 data. Statistical significance was defined by *p* value <0.05.

## Results

### Thirty-day readmission rate

A total of 4797 UC patients that underwent colectomy or proctectomy were included in the study. The 30-day readmission rate was 20% (*N* = 963).

### Univariate analysis

Table 1 is the univariate analysis of baseline patient characteristics, Table 2 is the univariate analysis of baseline patient laboratory results, and Table 3 is the univariate analysis of operative characteristics and complications. These tables summarize the differences in baseline characteristics between those who were and were not readmitted. Hispanic patients, patients with pre-existing congestive heart failure, hypertension requiring medication, or elevated alkaline phosphatase, patients undergoing proctocolectomy with IPAA creation and diverting ileostomy, patients with an unplanned return to the operative room during index admission, longer operative time, or certain post-operative complications had significantly higher rates of 30-day readmission. Table 4, which is the subgroup analyses by surgery performed of emergency admission and operative time, summarizes the results of evaluating emergency cases and operation time. Emergent surgery did not impact 30-day readmission rates on patients receiving abdominal colectomy with end ileostomy.

**Table 1:** Univariate analysis of baseline patient characteristics

	No 30-day readmission, *n* = 3834		30-Day readmission, *n* = 963		*p* value
	Number	%	Number	%	
*Gender*					0.65
Female	1655	43.2%	423	44.0%	
Male	2179	56.8%	539	56.0%	
*Race*					0.18
White	3276	93.8%	822	92.4%	
Black/African American	143	4.1%	49	5.5%	
Other^a^	74	2.1%	19	2.1%	
Hispanic	142	34.0%	60	6.6%	0.001
Age (years)	43.8	15.8	43.1	16.3	0.23
*Body mass index (kg/m* ^*2*^ *)* ^b^	26.2	5.8	26.4	6.0	0.39
Body mass index ≥30 (kg/m^2^)	846	22.3%	234	24.4%	0.16
Body mass index ≥40 (kg/m^2^)	91	2.4%	29	3.0%	0.27
Ascites^b^	10	0.3%	4	0.4%	0.50
*History of severe chronic*					
Obstructive pulmonary disease	38	1.0%	15	1.6%	0.13
Congestive heart failure in 30 days before surgery^c^	7	0.2%	6	0.6%	0.03
Hypertension requiring medication	693	18.1%	212	22.0%	0.005
Acute renal failure (pre-op)^c^	9	0.2%	1	0.1%	0.70
Steroid use for chronic condition	1963	51.2%	495	51.4%	0.91
>10% Loss of body weight in last 6 months	367	9.6%	82	8.5%	0.31
Bleeding disorders	132	3.4%	42	4.4%	0.17
Current smoker within 1 year	276	7.2%	78	8.1%	0.34
Transfusion prior to surgery	145	3.8%	31	3.2%	0.41
*American Surgery Association* *Classification*					0.22
1	75	2.0%	9	0.9%	
2	2217	57.9%	547	56.8%	
3	1442	37.7%	383	39.8%	
4	90	2.4%	22	2.3%	
5	6	0.2%	2	0.2%	

^a^Includes: Asian, American Indian or Alaska Native, and Native Hawaiian or Pacific Islander

^b^Variable log-transformed for analysis

^c^Fisher’s exact test

**Table 2:** Univariate analysis of baseline patient laboratory results

	No 30-day readmission, *n* = 3834		30-Day readmission, *n* = 963		*p* value
	Number	%	Number	%	
Pre-operative hematocrit	37.0	6.1	37.1	6.1	0.94
Pre-operative serum albumin	3.5	0.9	3.3	0.3	0.54
Pre-operative aspartate aminotransferase^a^	23.5	39.6	24.7	45.3	0.76
Pre-operative alkaline phosphatase^a^	85.0	70.6	89.8	75.3	0.02

^a^Variable log-transformed for analysis

**Table 3:** Univariate analysis of operative characteristics and complications

	No 30-day readmission, *n* = 3834		30-Day readmission, *n* = 963		*p* value
	Number	%	Number	%	
*Surgery*					
Open versus laparoscopic					0.80
Open	1793	46.8%	446	46.3%	
Laparoscopic	2041	53.2%	517	54.0%	
*Surgery performed*					<0.001
Abdominal colectomy with end Ileostomy	1390	36.3%	285	29.6%	
Proctocolectomy and end ileostomy	560	14.6%	117	12.2%	
Proctocolectomy with IPAA creation and diverting ileostomy	1000	26.1%	325	33.8%	
Proctectomy^a^	884	23.1%	236	24.5%	
Emergency case	208	5.4%	40	4.2%	0.11
Operation time (min)^b^	244.9	104.0	260.2	111.1	<0.001
Return to operating room	125	3.3%	140	14.5%	<0.001
Length of stay (days)^b^	8.7	8.9	8.3	5.9	0.67
*Complications*					
Surgical site infection^c^	368	9.6%	313	32.5%	<0.001
Wound disruption	35	0.9%	26	2.7%	<0.001
Pneumonia	54	1.4%	16	1.7%	0.56
Pulmonary embolism	19	0.5%	18	1.9%	<0.001
Renal insufficiency	18	0.5%	32	3.3%	<0.001
Urinary tract infection	109	2.8%	75	7.8%	<0.001
Cerebral vascular accident/stroke with neurological deficit^d^	2	0.1%	1	0.1%	0.49
Cardiac arrest requiring cardiopulmonary resuscitation/myocardial infarction^d^	10	0.3%	6	0.6%	0.11
Bleeding transfusions	479	12.5%	146	15.2%	0.03
Deep vein thrombosis/thrombophlebitis	81	2.1%	92	9.6%	<0.001
Sepsis/septic shock	179	4.7%	186	19.3%	<0.001

^a^Includes: (1) proctectomy, combined abdominal perineal pull-through, with creation of colonic reservoir, with diverting enterostomy, when performed (open surgery, laparoscopy surgery); (2) proctectomy, partial, with rectal mucosectomy, ileoanal anastomosis, creation of ileal reservoir, with or without loop ileostomy (open surgery)

^b^Variable log-transformed for analysis

^c^Includes: superficial surgical site infection, deep incisional surgical site infection, and organ space surgical site infection

^d^Fisher’s exact test

**Table 4:** Subgroup analyses by surgery performed of emergency admission and operation time

	No 30-day readmission		30-Day readmission		*p* value
Abdominal colectomy with end ileostomy	*n* = 1390		*n* = 285		
	Number	%	Number	%	
Emergency case^a^	175	12.60%	33	11.58%	0.694
Operation time (min)^b^	199.80	82.45	216.49	100.38	0.021

^a^Fisher’s exact test

^b^Variable log-transformed for analysis with *t* test

### Multivariate analysis for factors associated with readmission

Table 5, which is the multivariate logistic regression model for readmission within 30 days, summarizes risk factors associated with 30-day readmission. Patient’s ethnicity, such as Hispanic race (odds ratio (OR) 1.66, 95% confidence interval (CI) (1.11, 2.50)), and certain pre-existing conditions before surgery, such as congestive heart failure (OR 4.76, 95 % CI (1.09, 20.75)), lower albumin (OR 0.81, 95% CI (0.70, 0.93)), hypertension requiring medications (OR 1.43, 95% CI (1.12, 1.82)), and utilization of corticosteroids (OR 1.27, 95% CI (1.02, 1.57)) significantly impacted 30-day readmission rate in UC patients undergoing colectomy and proctectomy.

**Table 5:** Multivariate logistic regression model for readmission within 30 days

	OR	95% CI	*p* value
*Potentially modifiable*			
DVT/thrombophlebitis	5.69	(3.67, 8.83)	<0.001
Congestive heart failure in 30 days before	4.76	(1.09, 20.75)	0.038
Surgery			
Pulmonary embolism	4.34	(1.72, 10.91)	0.002
Return to OR	3.71	(2.56, 5.40)	<0.001
Renal insufficiency	3.38	(1.51, 7.54)	0.003
Surgical site infection	3.38	(2.59, 4.42)	<0.001
Urinary tract infection	2.83	(1.86, 4.31)	<0.001
Sepsis/septic shock	2.22	(1.57, 3.12)	<0.001
*Surgery*			0.003
Proctocolectomy and end ileostomy	Reference		
Abdominal colectomy with end Ileostomy	1.05	(0.76, 1.46)	
Proctocolectomy with IPAA formation and diverting ileostomy	1.53	(1.10, 2.10)	
Proctectomy^a^	1.69	(1.18, 2.42)	
Hypertension requiring medication	1.43	(1.12, 1.82)	0.004
Steroid use for chronic condition	1.27	(1.02, 1.57)	0.032
Every 30-min increase of operation time	1.04	(1.01, 1.07)	0.009
Every 1-day increase of length of stay	0.93	(0.91, 0.95)	<0.001
Every 1 g/dl increase of pre-operative serum album	0.81	(0.70, 0.93)	<0.001
*Not modifiable*			
Hispanic	1.66	(1.11, 2.50)	0.014

The area under the ROC curve was 0.734

^a^Includes: proctectomy, combined abdominal perineal pull-through, with creation of colonic reservoir, with diverting enterostomy, when performed (open surgery, laparoscopy surgery); proctectomy, partial, with rectal mucosectomy, ileoanal anastomosis, creation of ileal reservoir, with or without loop ileostomy (open surgery)

Type of surgery and other factors associated with surgery also significantly impacted 30-day readmission rates. Patients were more likely to be readmitted if they underwent proctocolectomy with IPAA creation (OR 1.53, 95% CI (1.10, 2.10)) or other surgeries including proctectomy with combined abdominal perineal pull-through with creation of colonic reservoir and diverting enterostomy, and proctectomy, partial with rectal mucosectomy, ileoanal anastomosis, and creation of ileal reservoir, with or without loop ileostomy (OR 1.69, 95% CI (1.18, 2.42)). Return to the operation room (OR 3.71, 95% CI (2.56, 5.40)), longer operation time (OR 1.04, 95% CI (1.01, 1.07)), and shorter LOS (OR 0.93, 95% CI (0.91, 0.95)) were associated with an increased risk of 30-day readmission. Post-operative complications including surgical site infection (OR 3.38, 95% CI (2.59, 4.42)), pulmonary embolism (OR 4.34, 95% CI (1.72, 10.91)), renal insufficiency (OR 3.38, 95% CI (1.51 7.54)), UTI (OR 2.83, 95% CI (1.86, 4.31)), DVT/thrombophlebitis (OR 5.69, 95% CI (3.67, 8.83)), and sepsis/septic shock (OR 2.22, 95% CI (1.57, 3.12)) were associated with higher 30-day readmission rates.

### Model performance

The ability of the logistic regression model to discriminate between patients who will be readmitted within 30 days and those who will not was evaluated with the area under the receiver operating characteristic curve (AUC). The AUC for the model fit with the developmental dataset was 0.73 (95% CI (0.71, 0.76)) demonstrating good discrimination. When the derived model is applied to the validation dataset, the discrimination of the model remains good with a similar AUC of 0.71 (95% CI (0.66, 0.75)).

### Risk score to predict readmission

**Fig. 1:**
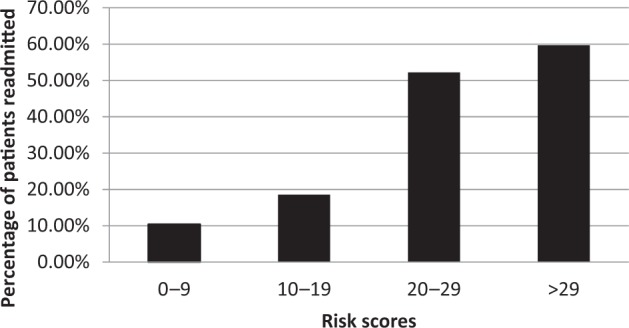
The proportion of patients were readmitted within 30 days based on the readmission risk scores

Table 6 outlines a risk score formulated in order to determine an individual’s risk for readmission. The range of risk score in the study was 0–58. Figure 1 describes proportion of patients readmitted. Ten percent of patients with a risk score between 0 and 9 were readmitted, 18.5% with a score between 10 and 19, 52.2% with a score between 20 and 29, and 59.6% in patients with a risk score >29. The average risk score for patients included in the study was 11.8 with a standard deviation of 8.53. In Table 7, the model is validated with NSQIO 2015 data. When the model is applied to the validation dataset, a similar trend was observed. The average risk score for patients was 11.7 with a standard deviation of 8.15.

**Table 6:** Risk score for readmission

	Weight
*Potentially modifiable*	
DVT/thrombophlebitis	15
Pulmonary embolism	13
Renal insufficiency	12
Surgical site infection	11
Return to OR	12
Congestive heart failure in 30 days before surgery	10
Urinary tract infection	9
Sepsis/septic shock	7
Proctocolectomy with IPAA creation and diverting ileostomy	5
Other surgeries^a^	5
Length of stay <7 days	4
Hypertension requiring medication	3
Steroid use for chronic condition	2
Pre-operative serum albumin <3.5 g/dl	2
Operation time >240 min	
*Not modifiable*	
Hispanic	5

^a^Proctectomy, combined abdominoperineal pull-through, with creation of colonic reservoir, with diverting enterostomy, when performed (open surgery, laparoscopy surgery) and proctectomy, partial, with rectal mucosectomy, ileoanal anastomosis, creation of ileal reservoir, with or without loop ileostomy (open surgery)

**Table 7:** Validation of the model with NSQIP 2015 data

	2011–2014		2015	
Risk score	Number of patients	Proportion readmitted	Number of patients	Proportion readmitted
0–9	170	10.39%	71	13.35%
10–19	186	18.54%	55	17.74%
20–29	166	52.20%	56	53.33%
>29	99	59.64%	23	57.50%

When the model shown in Table 3 is applied to the NSQIP 2015 data, the area under the ROC curve (AUC) is 0.709

## Discussion

UC is a chronic relapsing disease. Once patients have medically refractory UC, untoward side effects from medications, or develop dysplasia or malignancy, surgery is indicated. In this study, utilizing a large national clinical database, 20% of UC patients undergoing colectomy or proctectomy were readmitted within 30 days. While multiple factors were associated with readmission, factors with the highest association were potentially modifiable, including post-operative complications and uncontrolled pre-existing conditions. This study also established a risk score model to predict readmission in UC patients that undergo colectomy or proctectomy; therefore, by applying to each UC patient undergoing colectomy or proctectomy, the model will be able to determine patients with high risk of readmission. By targeting modifiable risk factors, healthcare providers might be able to reduce the 30-day readmission.

In our study, multiple pre-existing conditions of were associated with readmission. Hispanic patients with UC that undergo colectomy or proctectomy were more likely to be readmitted within 30 days. Hispanic patients with UC undergoing surgery have a higher risk of post-operative complications, and are more likely to be readmitted after surgery^14^. However, our study controlled for many post-operative complications, suggesting that other factors also contribute to the high 30-day readmission rate. Hispanic patients with UC more likely have increased disease severity and decreased use of biologics, which might partially contribute to the increased readmission after colectomy or proctectomy^15^.

UC patients with lower pre-surgical serum albumin, indicating malnutrition, were more likely to be readmitted. The impact of malnutrition on surgical outcomes has been well studied and is associated with increased complications, mortality, and readmission in abdominal surgery^16^; therefore, if surgery is not emergent, nutrition should be optimized prior to surgery to reduce 30-day readmission rates in UC patients.

Pre-existing heart failure and hypertension requiring medication were also associated with an increased risk of readmission in UC patients undergoing colectomy or proctectomy. Patients with heart failure that undergo non-cardiac surgery are at a high risk of complications, with 30% of patients experiencing myocardial infarction, exacerbation of pre-existing heart failure, or other complications that would prompt readmission^17^. In addition, patients with hypertension requiring medications were more likely have an unplanned readmission after receiving plastic surgery^18^. UC patients with heart failure and hypertension should be optimized before undergoing surgery to reduce the risks of readmission.

Chronic use of corticosteroids in patients with UC is associated with an increased risk of readmission, which may be related to an increased risk of post-operative complications and infections^19^. However, our study controlled for these variables, suggesting that other factors including increased disease severity in patients with chronic corticosteroid use may contribute to this as well. Every effort should be made to minimize corticosteroid use prior to surgery in UC patients to reduce the risk of 30-day readmission.

While multiple surgical techniques can be utilized to treat UC patients, these procedures can be broadly categorized into restorative and non-restorative procedures. Restorative procedures involve creation of a small intestinal pouch with restoration of natural bowel function, whereas non-restorative procedures involve creation of a permanent colostomy. Since restorative procedures involve creation of small intestinal reservoir and a low pelvic anastomosis, these procedures entail a higher degree of technical complexity and longer operative times compared to non-restorative procedures. Additionally, restorative procedures are associated with increased complications when compared to other types of colectomy^20^. As expected, our data indicated that restorative surgeries were associated with increased 30-day readmissions^21^. Though patients that undergo an abdominal colectomy with ileostomy can have complications, such as delayed wound healing^2^, patients that undergo proctocolectomy with IPAA creation have approximately a 20% chance of bowel obstruction and 5% chance of pelvic sepsis, which leads to an increased risk of readmission^20^. Given the high risk of complications associated with restorative procedures, many centers are now performing these surgeries in three stages to allow cessation of immunosuppression before the creation of intestinal pouch. Furthermore, given the technical complexity and complicated post op care of these patients, these procedures are best performed at high volume, specialized centers to minimize post-operative complications and resultant readmission^22^.

UC patients that experienced an unplanned return to the operating room during their index admission were more likely to be readmitted within 30 days and this trend has also been noted in patients that have undergone other surgeries, for instance, lower extremity arterial bypass surgery^23^. Longer operation time was associated with increased risk of readmission in UC patients undergoing colectomy or proctectomy in our study, which is in agreement with a prior study in patients that undergo plastic surgery^24^. Previous studies have noted an exponentially increased risk of complications as surgery length of time increased by 1–3 h intervals^24^.

UC patients, especially patients who have multiple comorbidities or fail to respond to medical therapy, are at an increased risk of post-operative complications after colectomy or proctectomy^9^. Post-operative complications were associated with increased risk of readmission not only in UC patients undergoing colectomy or proctectomy, but also in other patients undergoing other surgeries^25^. Given the increased financial burden of post-operative complications and 30-day readmission, healthcare providers should aim to reduce short-term complications to improve patient and hospital outcomes, including minimizing intraoperative fecal contamination, optimal DVT prophylaxis, and early removal Foley catheters following surgery^26,27^.

Shorter LOS was also associated with an increased risk for 30-day readmission in patients with UC that undergo colectomy or proctectomy. Similarly, patients with other chronic diseases, including heart failure and COPD with decreased hospital LOS have increased 30-day hospital readmission rates^28,29^. Previous studies on patients receiving care at the Veterans Association (VA) hospital projected that for each day, hospital LOS was reduced, there was a 6% chance of readmission^28^. The association of increased hospital 30-day readmission rate with decreasing index hospital LOS raises concerns about quality of care that may be affected by the interventions that have been instituted to reduce hospital costs and LOS^30^. While minimizing the economic burden of healthcare in society is crucial, focus must also be placed on the quality of care and safe discharge planning, with initiative such as the Enhanced Recover After Surgery, which has successfully decreased LOS, mortality, and readmission rates^31^.

This study has multiple strengths. Recognizing risk factors that are associated with readmission are crucial in order to determine interventions that can reduce this vulnerable patient population from unnecessary readmissions. We established the first risk score model in order to identify UC patients who are at the highest risk of readmission after colectomy or proctectomy. Utilizing this model will allow physicians to target and intervene on patients. This will not only improve hospital outcomes but patient outcomes. In addition, this knowledge will help tremendously in setting patient expectations and perioperative planning. Utilization of the ACS NSQIP database allows the collection of data on a large number of UC patients who underwent colectomy or proctectomy, which could not be completed from a single medical center study. Finally, this database includes a diverse patient population from different geographic regions of the United States allowing for an accurate representation of the entire country.

However, this study has several limitations. Due to the cross-sectional analysis utilized in this study, only association between risk factors and readmission rates could be determined. However, in order to determine causality, a prospective analysis must be performed. In addition, this study was unable to determine whether the patient was taking other medications, including immunomodulators and biologic medicines prior to surgery, which could also impact 30-day readmission. These medications are associated with immunosuppression, renal dysfunction, and other complications, which would make patients taking these medications more prone to develop specific complications, including infections and acute kidney injury; therefore these patients would have a higher risk of readmission^32^. This study also does not evaluate initiatives such as enhanced recovery, infection prevention bundles, extended venous thromboembolism prophylaxis or readmission reduction pathways, which have been shown to reduce readmission in other patients^31^.

This study utilized a large national clinical database to determine factors that are associated with 30-day readmission in patients with UC that undergo colectomy or proctectomy. Pre-existing conditions, corticosteroid use, and post-surgical complications were associated with increased 30-day readmission rates; therefore, optimization of the patients’ chronic medical conditions, nutritional status, medication, and prevention of post-surgical complications may decrease rates of readmission. Patients with UC undergoing colectomy or proctectomy might benefit from receiving staged surgeries at a tertiary care center with skilled surgeons to reduce readmissions. Focus on quality of care while decreasing LOS should be emphasized in order to decrease the 30-day readmission rates. Many factors associated with readmission are potentially modifiable and these should be targeted to improve readmission rates in this vulnerable patient population. Overall, 30-day readmissions have a major effect on patients, providers, and healthcare costs, and further research should be conducted to determine ways to reduce readmission rates.

## Study Highlights

### WHAT IS CURRENT KNOWLEDGE

- Thirty-day hospital readmission in patients undergoing surgery has a significant economic burden on the healthcare system.
- Approximately 5–20% of patients with ulcerative colitis (UC) undergo colectomy or proctectomy within 10 years of diagnosis and are at an increased risk of complications prompting unplanned hospital readmission.

### WHAT IS NEW HERE

- The 30-day readmission rate was 20% in UC patients receiving colectomy or proctectomy.
- There are multiple potentially modifiable risk factors that are associated with high 30-day readmission rate.

### TRANSLATIONAL IMPACT

- Intervention aimed at these risk factors may improve outcomes of these patients.

## Electronic supplementary material

Supplementary Table 1
