# Supplementary material for: A risk score model of 30-day readmission in ulcerative colitis after colectomy or proctectomy
Source: Clin Transl Gastroenterol. 2018 Aug 15;9(8):175. doi: 10.1038/s41424-018-0039-y (PMC6092348; doi:10.1038/s41424-018-0039-y)
Supplement: Supplementary file 1 — Supplementary Table 1 [file 41424_2018_39_MOESM1_ESM.docx]

**Supplementary Table 1 – Missing Data**

|  | Full Cohort  n=4,797 | |
| --- | --- | --- |
| Gender | 1 | 0.02% |
| Race | 414 | 8.63% |
| Hispanic | 316 | 6.59% |
| Body Mass Index | 33 | 0.69% |
| ASA Classification | 4 | 0.08% |
| Pre-operative hematocrit | 244 | 5.09% |
| Pre-operative serum albumin | 1,431 | 29.83% |
| Pre-operative AST | 1,610 | 33.56% |
| Pre-operative alkaline phosphatase | 1,555 | 32.42% |
| Emergency Case | 2 | 0.04% |
| Operation Time | 12 | 0.25% |
| Length of Stay | 2 | 0.04% |
